# Supplementary material for: A blockchain-based multi-authority hierarchical attribute encrypted data sharing scheme in the Internet of Medical Things
Source: PLoS One. 2026 May 27;21(5):e0349767. doi: 10.1371/journal.pone.0349767 (PMC13215620; doi:10.1371/journal.pone.0349767)
Supplement: S1 Appendix — All code used in this study is available in the following public GitHub repositories. • MIRACL Main Repository: https://github.com/mirac1/MIRACL. • MIRACL Core Library: https://github.com/mirac1/core. (PDF) [file pone.0349767.s001.pdf]

All code used in this study is available in the following public GitHub repositories:
MIRACL Main Repository:https://github.com/miracl/MIRACL.
MIRACL Core Library:https://github.com/miracl/core.
