# Supplementary material for: A blockchain-based multi-authority hierarchical attribute encrypted data sharing scheme in the Internet of Medical Things
Source: PLoS One. 2026 May 27;21(5):e0349767. doi: 10.1371/journal.pone.0349767 (PMC13215620; doi:10.1371/journal.pone.0349767)
Supplement: Attachment — Submitted filename: Reviews - PONE-D-25-37401_reviewer- FOR MULTI PARTY.pdf.docx [file pone.0349767.s003.docx]

**Summary**

The manuscript proposes **BMAHDS**, a blockchain-based, multi-authority CP-ABE scheme tailored to IoMT. Core ingredients are: (i) a **hierarchical access tree** that allows “batch” handling of multiple files with nested sensitivity levels; (ii) **multi-authority** key generation with a DKG flavor to avoid single points of failure and resist collusion; and (iii) **lightweight data-origin authentication** via BLS threshold signatures, with hashes and aggregate signatures recorded on-chain, while encrypted payloads live off-chain. The paper claims CPA security (under DBDH), collusion-resistance, and better storage/compute costs than recent baselines, reporting results from a JPBC/Java implementation on Type-A pairings (≈512-bit field, 160-bit order) and showing comparative plots of keygen, enc/dec, and ciphertext size versus number of attributes/files.

**Strengths**

1. **Problem fit to IoMT**: The hierarchical access idea matches clinical data stratification (e.g., labs < imaging < full EHR) and is clearly motivated.
2. **System sketch is coherent**: Off-chain encryption + on-chain hash/BLS aggregates is a sensible split for auditability without blockchain bloat.
3. **Attempt at full stack**: The paper connects cryptography (CP-ABE), distributed trust (multi-authority/DKG), and data-origin proofs (BLS threshold signatures) into one pipeline with verification equations spelled out.
4. **Comparative positioning**: A table maps features across prior work (hierarchy, multi-authority, collusion, blockchain, data-source authentication, verification).

**Major concerns**

1. **Security level / curves and fairness of comparisons**
   Experiments use **Type-A pairings over ~512-bit fields with ~160-bit group order**, which is well below commonly accepted contemporary security targets; BLS on super-singular Type-A groups is especially unusual today. Many baselines in 2022–2024 likely reported with stronger curves/parameters. Authors should either (a) re-run **all schemes** under the **same security level** (e.g., 128-bit, BLS12-381 or BW6-761) or (b) make a compelling argument that every comparison used the same weakened setting. As written, the absolute times and even **relative rankings** may change at modern security levels.
2. **Threat model and DKG/threshold assumptions are underspecified**
   The text mentions DKG and t-of-n verification by “assistant nodes,” but the **adversary model** for those nodes is not crisply stated: what fraction can be Byzantine? Are there **dishonest-majority** guarantees? How are **key shares** protected against compromise and **share refresh** handled? How is **Sybil resistance** or **identity binding** for assistant nodes achieved? Please formalize the threat model and the trust/availability assumptions for DKG and for the BLS aggregation network.
3. **Collusion-resistance and CPA proof clarity**
   The paper states CPA security under DBDH with resistance to multi-authority collusion, but the proof sketch is difficult to follow and seems **selective-policy** flavored. Please (i) state the **exact security model** (selective vs. adaptive; policy-hiding or not; corrupt authority capabilities), (ii) cleanly separate **user collusion** vs. **authority collusion**, and (iii) tighten notation to make the reduction steps traceable (right now, symbols like ψk\psi_kψk, seeds for PRFs across corrupt authorities, and the challenger’s distribution blend together). A clearly structured theorem/experiment interface (Init, Query, Challenge, Guess) would help.
4. **Blockchain layer is a black box**
   The manuscript never specifies chain type (public/consortium), consensus (e.g., PoS/BFT), **transaction cost/latency**, block size limits, or **throughput** vis-à-vis the expected telemetry rate of IoMT devices. Without even a back-of-the-envelope **gas/storage cost** and **latency** analysis (hash + threshold signature per batch), it’s hard to assess feasibility at hospital scale. The design relies on on-chain audits for integrity trails but provides no **operational evaluation** of this layer. Please add a concrete instantiation (e.g., Fabric/Quorum/ETH-testnet) and measure end-to-end verification latency.
5. **Revocation and dynamics**
   Attribute/key/user **revocation** is critical in healthcare; the paper acknowledges future work, but some minimal design (time-bounded attributes, epoch keys, or verifier-local revocation) is expected for technical sufficiency. Experiments should also explore **policy updates** and **user leave/join** costs.
6. **Evaluation design**
   *Worst-case access structures* are modeled as pure AND gates; real policies will mix AND/OR/k-of-n and deeper trees. Please include **mixed thresholds** and deeper hierarchies. Also clarify how you computed **KB** of ciphertexts (values of L1,L2,LpL_1,L_2,L_pL1,L2,Lp) and provide the **exact encodings** used. Several comparative claims (e.g., ciphertext ≈14.34 KB vs others up to ~75.65 KB at 50 attributes) need reproducible parameter tables and scripts. Finally, include **confidence intervals** or variance, not just means.

**Questions the authors should answer**

1. What **exact security level** (bits) do your Type-A parameters target, and how do you normalize comparisons against works using prime-order curves (e.g., BN/BLS12)?
2. What are the **t** and **n** values for the BLS threshold, and what failure/attack scenarios are covered (assistant dropouts, equivocation, rogue-key attacks)?
3. Is the scheme **policy-hiding**? If not, what is revealed by the access tree on the ciphertext? (Plausible in healthcare, but disclosure should be explicit.)
4. How are **user identifiers (GID)** handled to avoid cross-authority linkability while still preventing user collusion? (Some prior MA-ABE schemes struggle here.)
5. What is the **key/attribute revocation** path today (even if basic), and what is the expected cost in your hierarchy?

**Recommendation**

**Major Revision**. The idea is promising and relevant, and the paper is close to PLOS ONE’s “technically sound” bar. However, the **security level mismatch**, **under-specified threat model**, **unclear proof structure**, and **missing blockchain-layer evaluation** need to be addressed before publication.
