# Supplementary material for: A blockchain-based multi-authority hierarchical attribute encrypted data sharing scheme in the Internet of Medical Things
Source: PLoS One. 2026 May 27;21(5):e0349767. doi: 10.1371/journal.pone.0349767 (PMC13215620; doi:10.1371/journal.pone.0349767)
Supplement: Attachment — Submitted filename: Response to Reviewers.docx [file pone.0349767.s005.docx]

Dear Editors and Reviewers:

Thank you for giving us the opportunity to revise our manuscript. We sincerely appreciate the time and effort you have dedicated to providing insightful feedback on our work. We have carefully considered all comments and suggestions and have made revisions to the manuscript accordingly.Our point-by-point responses to the comments are listed below.

**Responses to comments from Reviewer#1**

**Comment 1:**Clarity of Explanation: While the technical contributions are substantial, some sections, especially those describing the hierarchical access tree construction and the mechanics of the DKG and co-signature schemes, could benefit from clearer, step-by-step explanations or illustrative diagrams. This would aid readers in fully understanding the novel mechanisms and their interactions.

**Response:** We sincerely appreciate your recognition of our work and the valuable suggestions you have provided. Your insightful comments regarding the need for more precise step-by-step explanations and illustrative diagrams to elaborate on the hierarchical access tree, DKG, and cooperative signature mechanism have been crucial in enhancing the readability of our paper. Based on your feedback, we have made substantial revisions to the manuscript:

1. For the hierarchical access tree, we have provided a detailed explanation in Section 3.4 of Chapter 3.

2. For the DKG cooperative signing section, we have rewritten Section 5.1 into a logically more straightforward four-phase workflow (registration, DKG protocol flow, signing process, key update), highlighting the complete trust chain from “node identity binding” to “distributed key generation” to “cooperative data authentication.” Additionally, we have specifically added Figure 4 (DKG Protocol Flowchart) and Figure 5 (Complete Data Sharing Interaction Diagram). These sequence diagrams clearly illustrate the message exchange sequence and specific content between IoMT devices, assistant nodes, the blockchain, and data owners. They intuitively reveal how these mechanisms interact to achieve distributed trust and secure data traceability jointly.

3. Finally, we would like to express our sincere gratitude once again for your invaluable assistance in significantly enhancing the quality and expressiveness of our paper!

**Comment 2:**Comparative Analysis: The paper references multiple existing schemes and discusses their limitations. Including a more detailed comparative quantitative analysis—such as benchmarks or simulation results—would better illustrate how the proposed scheme outperforms existing solutions across various metrics like encryption/decryption times, storage overhead, and security levels.

**Response:** Dear Reviewer, We sincerely appreciate your thorough review of our work and your valuable suggestions. We fully agree that a detailed analysis incorporating quantitative comparisons and benchmarking is essential for clearly demonstrating the performance advantages of the proposed scheme. Following your recommendations, we have made the following significant additions and modifications in the revised manuscript to provide a more comprehensive and persuasive performance comparison:

1. First, we have systematically expanded the theoretical analysis in Section 7.1 to include quantitative comparisons with existing schemes such as ([13], [15], [16], [26], [27]), and detailed the theoretical expressions for storage overhead (authoritative public keys, master keys, user private keys, ciphertext) in Table 4. We particularly highlight the cryptographic curves relied upon by different schemes (Type-1 symmetric pairing versus the Type-3 asymmetric pairing adopted in our scheme) and their corresponding security strengths.

2. Additionally, Table 5 has been added to compare the computational costs of user revocation, user addition, and policy updates. Furthermore, Section 7.2 (Experimental Analysis) supplements the results with extensive simulation experiments. For encryption/decryption overhead, we tested the performance of our scheme against the comparison schemes under scenarios of increasing file counts (Figures 6c and 6d) and increasing attribute counts (Figures 6e and 6f). The new Figure 6a provides a visual comparison of ciphertext storage overhead.

3. Furthermore, we specifically address your concerns regarding security levels. In our experiments, we implemented both Type-1 symmetric pairing and BLS12-381 curve (Type-3 asymmetric pairing) schemes. The newly added Figure 7 clearly demonstrates the performance of these two approaches for user revocation, user addition, and policy update operations. Experimental results demonstrate that our scheme achieves a higher security level (128 bits) while delivering significantly superior computational performance compared to symmetric pairing Type-1 curves, thanks to the efficiency of the BLS12-381 curve. This makes it better suited for the security requirements of modern medical IoT applications.

4. Finally, we extend our sincere gratitude once again for your invaluable assistance in refining this work!

**Comment 3:**Security Evaluation: The security analysis is promising but could be strengthened by more formal proofs or models, especially regarding resistance to collusion among multiple authorities and the potential for adversarial behavior within the blockchain network.

**Response:** Dear Reviewer, We sincerely appreciate your affirmation of our work and the insightful suggestions you have provided. We fully agree that a more formalized security model and proof would significantly enhance the rigor and persuasiveness of the security analysis in our paper. Based on your valuable feedback, we have revised the security model and security analysis in the revised manuscript as follows:

1. We have reaffirmed and refined the adversary model defined in Section 4.2. This model explicitly stipulates that in a system comprising N authorities, the adversary can compromise at most N-2 authorities, meaning at least two authorities must remain honest. This directly simulates the security boundary the system must maintain under the worst-case scenario involving malicious internal nodes. Since the scheme employs a pseudorandom function based on anonymous key exchange to construct user keys, the secret seeds held by at least two honest authorities prevent the adversary from forging a complete user private key that can pass decryption verification. We prove that this security property holds under the DBDH assumption through a rigorous reduction.

2. Furthermore, following the proof of Theorem 1, we have added a dedicated section on resistance to authority collusion attacks, explaining in more intuitive terms the ability to withstand N-1 corrupted authorities. Furthermore, to address "potential adversarial behavior in blockchains," we introduce Definition 2 in Section 4.2 (Security Models) to explicitly specify the formal security objectives that our proposed blockchain-assisted DKG protocol must satisfy: validity, robustness, confidentiality, and resistance to Sybil attacks. Theorems 2 and 3 formally prove the validity and robustness of the DKG protocol, respectively, ensuring correct execution even in the presence of Byzantine nodes. Theorem 4 formally proves confidentiality, demonstrating that adversaries cannot obtain the master private key without reaching the threshold. Theorem 5 specifically addresses resistance to Sybil attacks and Byzantine behavior, leveraging the blockchain's economic model to ensure the trustworthiness of the node set.

3. Finally, we extend our gratitude once again for your invaluable assistance in making this work more compelling!

**Comment 4:** Practical Deployment Considerations: The paper would benefit from a discussion of real-world implementation aspects, such as computational load on resource-constrained IoMT devices, potential network latency issues, and strategies for key revocation and update in dynamic environments.

**Response:** We sincerely appreciate your valuable and practical feedback. We fully agree that the practical value of a solution hinges on its consideration of real-world challenges. Based on your suggestions, we have further strengthened the discussion in these areas in the revised draft, as detailed below:

1. Regarding computational load on resource-constrained IoMT devices: As described in Section 5.1.3 (Signature Phase), IoMT devices only need to perform a single lightweight BLS signature (Equation 7), involving just one hash operation and one elliptic curve point multiplication. Computationally intensive tasks—such as CP-ABE encryption, threshold signature generation, and aggregation—are handled by data owners and assistant nodes.
2. Regarding "potential network latency issues," as described in Section 5.1.1, our DKG protocol is built upon an asynchronous network model and satisfies the Byzantine fault tolerance condition P≥3f+1. Furthermore, in the blockchain experiments presented in Section 7.2 (Figure 8c), we specifically evaluated the system's average latency in relation to transaction throughput. Results show that under loads ranging from 50 to 300 TPS, system latency remains below 280 milliseconds, demonstrating our solution's ability to deliver usable service performance in real-world environments with network latency.
3. Regarding "key revocation and update strategies in dynamic environments," we detailed a periodic revocation and update mechanism based on proxy re-encryption at the end of the data sharing phase (user revocation) in Section 5.2. The core advantage of this mechanism lies in offloading the computationally intensive task of updating ciphertexts from data owners to cloud service providers. This enables efficient, automated key rotation and policy updates, significantly reducing the overhead of dynamic system management. We particularly emphasize that when access policies require updates, data owners do not need to download and re-encrypt entire files. The algorithm only regenerates corresponding ciphertext components for newly added or modified attribute nodes. This update approach reduces communication and computational costs associated with dynamic policy adjustments.
4. Finally, we extend our sincere gratitude once again for helping us highlight the practical application value of this work!

**Comment 5:**Ethical and Data Privacy Implications: Given the sensitivity of medical data, explicit mention of compliance with relevant data protection regulations (e.g., HIPAA, GDPR), as well as considerations for patient consent and data anonymization, should be included to reinforce the scheme’s practical applicability and ethical compliance.

**Response:** Dear Reviewer, Thank you for bringing this critical point to our attention. We fully agree that strict adherence to ethical guidelines and data protection regulations is essential for any research protocol involving sensitive medical data. This is not only a legal requirement but also a prerequisite for building user trust and advancing the implementation of technology. Based on your suggestion, we have made the following modifications and clarifications in the paper to strengthen the protocol's ethical compliance and practical applicability:

1. Regarding compliance with data protection regulations (e.g., HIPAA, GDPR) and patient consent, we have clarified in the introduction that high-sensitivity requirements mandate stringent protection of patient privacy and adherence to mandatory provisions of data protection regulations such as HIPAA and GDPR. We further clarify that the adopted CP-ABE supports the GDPR's "data minimization" and "purpose limitation" principles, as well as the HIPAA "minimum necessary" standard. This is because it enables patients (data owners) to directly control 'who' can access their data under what conditions through granular access policies, serving as a robust mechanism for informed consent. Data remains encrypted at all times when access policies are not satisfied, ensuring confidentiality.
2. Regarding patient consent and data anonymization, within our system model, the data owner is the patient or their direct agent. As described in Section 4.1, the DO is responsible for defining data access policies. This action logically implies the process of patient authorization and consent. The scheme enforces the patient's access control intentions through technical means.
3. Regarding data anonymization, we appreciate your insightful comment. We wish to clarify in our response that this paper's research scope focuses on addressing "confidentiality" and "controlled access" issues during medical data storage and sharing—precisely the core strengths of CP-ABE technology. Data anonymization is typically employed for population-level analysis where individual traceability is unnecessary, representing a distinct yet complementary technical domain from the "fine-grained access control" problem addressed in this work. We acknowledge the importance of anonymization and will explore integrating this scheme as a foundational security layer with anonymization techniques in future research. Furthermore, all performance evaluations in this experiment were conducted using simulated data, without any real, personally identifiable medical data.
4. Finally, thank you once again for helping us better present the practical applicability of this work!

**Comment 6:**Some typographical errors and grammatical inconsistencies are present throughout the manuscript. A thorough proofreading is recommended.

**Response:** Dear Reviewer, We sincerely appreciate your meticulous review of our manuscript and your valuable feedback on the formatting and grammatical issues identified. We fully recognize the critical importance of linguistic accuracy and standardized formatting in academic communication, and we sincerely apologize for these oversights in the previous version. Following your recommendations, we have conducted a thorough and meticulous proofreading of the entire manuscript. Specific revisions include:

Reviewing and standardizing technical terminology, abbreviations, and incomplete reference information (missing volume/issue numbers), while correcting grammatical errors, spelling mistakes, and punctuation issues throughout the text.

Additionally, we refined and restructured specific unclear or verbose sentences to enhance fluency and readability. Furthermore, we ensured consistent formatting and accurate citations for all figures, tables, and their captions.

Finally, we extend our sincere gratitude once again for your invaluable assistance in elevating the overall quality of this paper. Your meticulous approach has been immensely beneficial to us!

**Comment 7:**Figures and diagrams illustrating the hierarchical access tree structure and key management processes would enhance comprehensibility.

**Response:**Dear Reviewer, thank you very much for your valuable suggestions! We fully agree that clear illustrations are crucial for helping readers quickly understand complex cryptographic mechanisms. Based on your recommendations, we have made the following revisions to the manuscript:

1. For the hierarchical access tree, we have provided a step-by-step explanation.
2. Regarding the key management process, we have specifically added Figure 4 (DKG Protocol Process) and Figure 5 (Data Sharing Process). Figure 4 uses a sequence diagram to clearly illustrate the complete interaction flow, message sequence, and verification steps between assistant nodes and between them and the blockchain within the Distributed Key Generation (DKG) protocol. Figure 5 illustrates the end-to-end data sharing interaction, from encryption and upload to decryption verification, providing an intuitive view of the interactions among multiple roles—data owners, cloud servers, users, and others—in key management and data flow.
3. Finally, we extend our sincere gratitude once again for significantly enhancing the readability and expressiveness of our paper!

**Comment 8:**Clarifying the assumptions about the trustworthiness of different authorities and nodes within the system is important for highlighting the security model.

**Response:** Dear Reviewer, Thank you for bringing this crucial point to our attention. We fully agree that clearly articulating the trust assumptions for different entities within the system is fundamental to defining the security model and evaluating the robustness of the solution. Following your suggestion, we have explicitly formalized all key assumptions in Section 4.2 (Security Model) of the paper, as detailed below:

1. Regarding the trust assumptions for Authority Agencies (AAs), we assume that in a system comprising N AAs, an adversary can corrupt at most (N-2) AAs. This means that at least two honest AAs must always remain, incapable of colluding with the adversary. This assumption ensures security during the encryption phase and guarantees the protection of selectively resistant plaintext. Resisting attacks by (N-1) corrupted AAs safeguards against collusive attacks among AAs.
2. Additionally, regarding the trust assumptions for blockchain assistant nodes, we assume that in a network of P assistant nodes, an adversary can control at most f Byzantine nodes capable of arbitrary malicious behavior. The system must satisfy the Byzantine fault tolerance condition P ≥ 3f + 1. Under these assumptions, the effectiveness, robustness, and confidentiality of the distributed key generation protocol and cooperative signature mechanism are ensured. As proven in Theorems 2, 3, 4, and 5, even with f malicious nodes present, an honest majority (H ≥ 2f + 1) guarantees the protocol's correct completion, generating valid keys and signatures while ensuring the DKG protocol's master private key remains undisclosed.
3. Additionally, the medical cloud service provider is modeled as an honest but curious semi-trusted entity. It will faithfully execute the protocol but may attempt to learn additional information from the data it stores or processes.
4. Finally, thank you once again for helping us strengthen the argumentation in this critical section!

**Reviewer #2: This is a very interesting article, the authors present a novel approach combining blockchain with multi-authority hierarchical attribute-based encryption. However, I believe there are still some shortcomings**

**Responses to comments from Reviewer#2**

**Comment 1:**At the end of the introduction, the research objectives are vague, please simplify.

**Response:**Dear Reviewer, thank you for bringing this critical issue to our attention. We agree that a clear and well-defined research objective is essential for guiding readers' understanding of the entire paper. Following your suggestion, we have made significant revisions to the introduction's conclusion. The specific changes are as follows:

1.We removed redundant background descriptions and transitional phrases from the original paragraph, consolidating and refining the research objective with the core contributions of this paper. The revised conclusion now presents the three core problems addressed and the four main contributions proposed in more direct, concise language, making them more prominent and easily grasped. The modified paragraph directly focuses on our solution's design objectives—overcoming the efficiency, validation, and centralization bottlenecks of traditional CP-ABE in IoMT healthcare data sharing scenarios—while briefly outlining the technical approach we adopted to achieve these goals.

2.Finally, thank you once again for helping us enhance the clarity and impact of our paper!

**Comment 2:**Please add more content about data source verification.

**Response:** Dear Reviewer, Thank you very much for your valuable suggestions! We fully agree on the importance of strengthening the references related to verifying the authenticity of data sources. Following your advice, we have specifically supplemented the paper with key literature closely related to data source authentication, including the following:

1. New references [29-31] have been added. These three papers provide robust theoretical support for data verification mechanisms from distinct perspectives. The matching encryption scheme proposed in [29] offers crucial cryptographic foundations for data source authentication. The attribute-based matching encryption scheme in [30] provides methodological insights for implementing data source verification under fine-grained access control. The data source verification approach in [31], which outsources computational tasks to edge nodes, shapes our future research direction. As explicitly stated in our conclusions in Section 8, we plan to offload computationally intensive tasks (attribute-based cryptographic operations and partial signature verification) to edge nodes to reduce system latency and further enhance the practicality and scalability of the scheme.

2. Finally, we extend our sincere gratitude once again for your guidance!

**Comment 4:**Based on the IoMT scenario, it is recommended to add content on real-time data and computing power.

**Response:** Dear Reviewer, Thank you very much for your valuable suggestions! We fully agree that it is crucial to thoroughly consider real-time data processing requirements and the computational limitations of edge devices in IoMT scenarios. Based on your recommendations, we have further strengthened the relevant content in the paper. The specific modifications and explanations are as follows:

1. In the introduction, we outlined the system requirements for high-concurrency, strongly real-time data stream processing in typical IoMT applications (e.g., continuous vital sign monitoring). We also highlighted the need for lightweight deployment of security protocols within the resource-constrained context of edge devices.
2. Additionally, during the signing phase, we demonstrate our hierarchical processing mechanism where IoMT devices perform only a single lightweight BLS signature, while computationally intensive threshold signature generation and aggregation are handled by relatively resource-rich assistant nodes.

3. In the data sharing phase, the proposed hierarchical access tree structure optimizes encryption processes by reducing redundant computations, thereby enhancing processing efficiency for data with hierarchical relationships.

4. In the blockchain-based DKG protocol, shifting traditional centralized verification to distributed collaborative computation effectively reduces computational burden on end devices. Performance evaluations in Section 7.2 demonstrate our solution maintains low computational overhead for critical operations like encryption and decryption, proving its suitability for resource-constrained environments. Blockchain experiments show the system maintains stable response performance under typical workloads, meeting real-time requirements.

5. Finally, thank you once again for your guidance!

**Reviewer #3: Major Revision. The idea is promising and relevant, and the paper is close to PLOS ONE’s “technically sound” bar. However, the security level mismatch, under-specified threat model, unclear proof structure, and missing blockchain-layer evaluation need to be addressed before publication.**

**Responses to comments from Reviewer#3**

**Comment 1:**Security level / curves and fairness of comparisons
Experiments use Type-A pairings over ~512-bit fields with ~160-bit group order, which is well below commonly accepted contemporary security targets; BLS on super-singular Type-A groups is especially unusual today. Many baselines in 2022–2024 likely reported with stronger curves/parameters. Authors should either (a) re-run all schemes under the same security level (e.g., 128-bit, BLS12-381 or BW6-761) or (b) make a compelling argument that every comparison used the same weakened setting. As written, the absolute times and even relative rankings may change at modern security levels.

**Response:** Dear Reviewer, we sincerely appreciate your insightful and professional feedback. You correctly point out that performance comparisons conducted under outdated safety parameters render conclusions invalid, and we fully concur with your perspective. We take this issue very seriously and wish to clarify the following:

1. In our experiments, we have strictly followed the path (a) you suggested, namely, comparing all schemes at the same security level. Specifically, in the experiments described in Section 7.2 (Experimental Analysis), we did not use the default weak parameters. Instead, all schemes involved in the comparisons were configured and run using parameters providing approximately 128 bits of security strength. For baseline schemes requiring Type-1 symmetric pairing, we achieved a security level of ~128 bits by increasing the size of the underlying field and group. For our own scheme, we similarly provide performance data under both Type-1 pairing and Type-3 pairing (BLS12-381 curve). Therefore, all performance metrics reported in our paper (including Figures 6, 7, and 8), along with the absolute times and relative rankings derived from them, are obtained under a unified, modern 128-bit security level. This ensures fair performance comparisons and reliable conclusions. Under Type-1 pairing, our scheme achieves a high security level that matches modern applications, while still demonstrating computational and storage efficiency advantages. Furthermore, when our scheme switches to the more efficient BLS12-381 curve, its performance improves significantly. Thus, the relative performance rankings and core conclusion drawn in our paper—that our scheme is more efficient—remain robust. This conclusion holds first in the fair Type-1 pairing comparison and is further strengthened under the more modern Type-3 pairing.

2. Finally, we extend our gratitude once more for prompting us to clarify this critical detail, thereby fortifying our argument and enhancing its persuasiveness!

**Comment 2:Threat model and DKG/threshold assumptions are underspecified**
The text mentions DKG and t-of-n verification by “assistant nodes,” but the **adversary model** for those nodes is not crisply stated: what fraction can be Byzantine? Are there **dishonest-majority** guarantees? How are **key shares** protected against compromise and **share refresh** handled? How is **Sybil resistance** or **identity binding** for assistant nodes achieved? Please formalize the threat model and the trust/availability assumptions for DKG and for the BLS aggregation network.

**Response:** Dear Reviewer, thank you very much for your valuable suggestions regarding the formalization and precision of the security model! We fully agree that clearly defining the adversarial models and trust assumptions for participants in distributed protocols is crucial. Following your advice, we have formally articulated these key assumptions and mechanisms in Section 4.2 (Security Models) and Section 5.1 (Blockchain-based DKG Protocol) of the paper. We report the following updates:

1. Regarding the adversarial model and Byzantine fault tolerance ratio: In Section 4.2's security model, we explicitly assume: within a system comprising P assistant nodes, there exists an adaptively adversary with computational bounds who can control at most f nodes exhibiting arbitrary Byzantine behavior. Its fault tolerance threshold adheres to standard BFT consensus conditions, requiring the total number of nodes to satisfy P ≥ 3f + 1. This implies the proportion of Byzantine nodes must remain below one-third. Under this condition, an honest majority (H ≥ 2f + 1) is guaranteed. This ensures that the protocol's validity, robustness, and confidentiality (proven by Theorems 2, 3, and 4) remain intact even in the presence of a dishonest minority. Once the proportion of malicious nodes exceeds 1/3, the system can no longer guarantee liveness and safety, consistent with classical BFT conclusions.
2. Confidentiality, Leak Protection, and Refresh Mechanism for Key Shares: After generation, each assistant node's key share $sk_{i}$ is immediately encrypted with its public key $PK_{i,enc}$, and the hash value is submitted to the blockchain for notarization. The original share is securely stored locally. Due to the DKG protocol's threshold mechanism, an adversary must collect t = 2f + 1 valid shares to reconstruct the master private key $SK_{group}$. Since an adversary can control at most f nodes, they cannot reach the required threshold. Furthermore, our scheme achieves forward secrecy through the periodic key share update mechanism described in Section 5.1.4. This mechanism updates shares by having all nodes jointly generate a new polynomial $\Delta f_{j}(x)$ with a constant term of zero (Formulas 14, 16). Consequently, even if an adversary obtains the old $sk_{i}$ and expired shares $sk'_{i}$, they cannot compute new valid shares SKB or the master private key, thereby ensuring the security of historical signatures.
3. Sybil Attacks and Identity Binding: We define this as a security property in Section 4.2 (Security Model) and implement it through technical means in Section 5.1.1 (Registration Phase). Each assistant node must register using its unique blockchain address and bind its signature key pair upon joining the network. Additionally, registration typically requires staking a certain amount of collateral into a smart contract. During protocol execution (e.g., steps 4-5 of DKG), any malicious behavior confirmed by the BFT consensus protocol (such as submitting invalid shares) will result in the node's collateral being automatically forfeited by the smart contract, and its registered identity will be revoked. This ensures that creating numerous fake identities incurs prohibitively high and unsustainable economic costs, thereby achieving resistance to Sybil attacks. With these additions, our paper now formally defines the threat model for DKG and BLS aggregation networks.
4. Finally, thank you once again for helping us strengthen the rigor of this critical section!

**Comment 3:Collusion-resistance and CPA proof clarity**
The paper states CPA security under DBDH with resistance to multi-authority collusion, but the proof sketch is difficult to follow and seems **selective-policy** flavored. Please (i) state the **exact security model** (selective vs. adaptive; policy-hiding or not; corrupt authority capabilities), (ii) cleanly separate **user collusion** vs. **authority collusion**, and (iii) tighten notation to make the reduction steps traceable (right now, symbols like ψk\psi_kψk, seeds for PRFs across corrupt authorities, and the challenger’s distribution blend together). A clearly structured theorem/experiment interface (Init, Query, Challenge, Guess) would help.

**Response:** Dear Reviewer, we sincerely appreciate your insightful and professional comments on the security proof section of our paper! We fully agree that a well-structured, rigorously formalized security model and proof are crucial for establishing the scheme's credibility. Following your valuable suggestions, we have made significant revisions to Section 4.2 (Security Model) and Section 6 (Security Analysis) to address all your concerns.

1. Clarification and Formalization of the Precise Security Model: We have formally defined a selective security model in Section 4.2 and clarified the following key points: (a) Security Objective: The scheme aims to achieve indistinguishability against a chosen plaintext attack. (b) Adversary Model: This is a selective security model. The adversary must declare in advance, during system initialization, the challenge access structures they intend to attack and the set of authorities they plan to corrupt. (c) Policy Hiding: The security model in Section 4.2 specifies that the scheme does not provide policy hiding; the challenge access structure is explicitly embedded in the challenge ciphertext. (d) Adversary Corruption Capability: The adversary can corrupt at most N-2 authorities, meaning at least two authorities must remain honest throughout the system.
2. Regarding the clear distinction between user collusion and collusion with authority: (a) User collusion resistance: Achieved by binding a global identifier (GID) to each user and introducing user-specific random factors during the key generation phase. During Phases 1 & 2 of the security game, an adversary may query private keys corresponding to multiple distinct attribute sets (representing different users). However, due to randomization, these private keys cannot be combined to decrypt a ciphertext that none of them could decrypt individually. (b) Authority Collusion Resistance: Two authorities, $A_{k}$ and $A_{j}$, cooperatively generate a pseudorandom function (PRF) seed $s_{kj}$, which remains confidential between them. Even with up to N-2 corrupt authorities, they cannot know these two PRF seeds. Furthermore, the private keys of the authorizing authorities are combined with the users' private keys. Consequently, even if $A_{k}$ and $A_{j}$exhibit malicious behavior, the adversary cannot obtain a valid key. Thus, the proposed scheme resists attacks from up to N-1 colluding authorities.
3. Symbolic Representation Refinement and Proof Structure Organization: (a) Symbolic Clarification: We have precisely defined all critical symbols in the symbol table and clearly distinguished parameters for different roles. (b) Structured Security Game and Reduction: We have rigorously restated the security game following the structure: Init → System Setup → Authority Setup → Phase 1—Key Query → Challenge → Phase 2—Key Query → Guess. In the proof of Theorem 1, we detail how the simulator, operating within this game framework, reduces the scheme's security to the DBDH assumption by transforming any adversary capable of winning with advantage ϵ into a DBDH problem solver. The proof guarantees that parameters generated by the simulator remain computationally indistinguishable from those in the real game at all stages.
4. Finally, thank you once again for helping us strengthen the rigor of this critical section!

**Comment 4:Blockchain layer is a black box**
The manuscript never specifies chain type (public/consortium), consensus (e.g., PoS/BFT), **transaction cost/latency**, block size limits, or **throughput** vis-à-vis the expected telemetry rate of IoMT devices. Without even a back-of-the-envelope **gas/storage cost** and **latency** analysis (hash + threshold signature per batch), it’s hard to assess feasibility at hospital scale. The design relies on on-chain audits for integrity trails but provides no **operational evaluation** of this layer. Please add a concrete instantiation (e.g., Fabric/Quorum/ETH-testnet) and measure end-to-end verification latency.

**Response:** Dear Reviewer, we sincerely appreciate your invaluable and efficient suggestions. We fully agree that the feasibility of a solution hinges on the specific implementation details and performance of its underlying system, such as blockchain. The issues you raised—including chain type, consensus mechanism, cost, and latency—are precisely the critical factors that bridge our theoretical framework to real-world applications. Based on your insightful feedback, we have made substantial additions and revisions to the paper, as detailed below:

1. Clarification of blockchain type, consensus mechanism, and key parameters: In Section 4.1 (System Model) and Section 7.2 (Experimental Analysis), we explicitly state that our solution employs a permissioned consortium blockchain. This architecture is well-suited for scenarios like medical data sharing, which require identity verification and multi-institutional collaboration. Additionally, we employ a Byzantine Fault Tolerant (BFT) consensus mechanism, explicitly setting the fault tolerance threshold to $t=[\frac{2}{3}n]$ in the BFT experiments detailed in Section 7.2. Our experimental configuration specifies a block size of 2 MB and a block interval of 2 seconds, forming the foundation for evaluating system throughput and latency.

2. Transaction Throughput, Latency, and Feasibility Analysis: (a) Throughput and Latency: We measured the system's average latency under loads ranging from 50 to 300 TPS. Results show that even under 300 TPS stress, average latency remained below 280 milliseconds. This provides direct quantitative evidence for evaluating the system's capability to process real-time data streams at a hospital scale. (b) Storage Overhead Analysis: We analyzed core on-chain storage components (e.g., hashes of DKG commitments, hashes of cryptographic key shares) and specifically highlighted (Figure 8(b)) that the BLS threshold signature length used in this scheme remains constant at 96 bytes—significantly lower than the on-chain storage overhead of traditional ECDSA signatures. This lightweight on-chain storage characteristic makes the storage cost of the scheme acceptable in consortium blockchain environments. Additionally, the transaction latency data (Figure 8(c)) and BFT authentication success rate data (Figure 9(a)) we provide collectively form the core components for evaluating this operational process. The former measures the efficiency of data reaching the chain and achieving consensus. At the same time, the latter assesses the robustness of the verification mechanism in maintaining a high success rate (>90%) even in the presence of malicious nodes (with a maximum Byzantine node ratio of 33%).

3. Additionally, as illustrated in Figure 9(b), we evaluated the system's average latency and transaction throughput (TPS) under increasing proportions of malicious nodes. Experimental results indicate that each 5% rise in malicious nodes leads to an approximate increase of 15–25 ms in consensus latency. When the malicious node ratio reached 30%—approaching the theoretical BFT fault tolerance limit of 33%—latency rose to 250 ms, indicating that the system preserves operational responsiveness even under significant disruption. Throughput exhibited a linear decline as the number of malicious nodes increased, falling from 470 TPS with no malicious nodes to 320 TPS at a 30% malicious node ratio. This quantifies the performance impact of adversarial conditions while confirming that the system maintains viable processing capacity under duress. Together with the authentication success rate depicted in Figure 9(a), these results comprehensively characterize the system's behavioral range from "high-performance" to "high-adversarial" scenarios. This evidence strongly substantiates the practical viability of our solution in real-world hospital settings, where node failures or malicious behavior may occur.

4. Finally, thank you once again for helping us strengthen the rigor of this critical section!

**Comment 5:Revocation and dynamics**
Attribute/key/user **revocation** is critical in healthcare; the paper acknowledges future work, but some minimal design (time-bounded attributes, epoch keys, or verifier-local revocation) is expected for technical sufficiency. Experiments should also explore **policy updates** and **user leave/join** costs.

**Response:** Dear Reviewer, thank you very much for your valuable feedback. We fully agree with your perspective that in a dynamic medical data sharing environment, an efficient and secure revocation mechanism is the cornerstone for the practical implementation of the scheme, not merely a prospect. We sincerely accept your critique and acknowledge that this aspect may not have been sufficiently highlighted in the initial version. In fact, our solution already incorporates a comprehensive revocation and update mechanism based on proxy re-encryption and periodic epoch identifiers. Following your recommendation, we have strengthened the exposition of this component in the paper, as detailed below:

1. Implemented Revocation Mechanisms: (a) Core Mechanism: We employ a hybrid model combining periodic key updates and proxy-based encryption. (b) Time Constraints and Periodic Keys: The system introduces an epoch identifier embedded within the system's public key, master key, and user private keys (Formulas 17, 18). This inherently implements an implicit time-bound property. When the system enters a new security epoch, old keys automatically expire, enabling periodic key rotation. (c) Verifier-Local Revocation: Through the epoch identifier mechanism, any participant verifying a key or ciphertext can rapidly determine revocation status by locally checking whether its epoch identifier is current. (d) Proxy Re-encryption: To reduce the burden on data owners, computationally intensive ciphertext updates are outsourced to cloud service providers. An authority generates a lightweight re-encryption key, which cloud servers use to automatically update old ciphertexts CT to new-cycle ciphertexts CT1. For non-revoked users, their keys are also synchronously updated to the new cycle by the cloud servers.
2. We have experimentally investigated the costs associated with policy updates and user dynamic join/leave operations: In Section 7.2 (Experimental Analysis), we quantify and compare the costs of revocation and update operations using Figure 7 and Tables 5 and 6. (a) Policy Update Cost: As described in user revocation (Step 4), when access policies require updating, data owners need not re-encrypt entire files. Instead, they only regenerate the corresponding ciphertext components $\tilde{C}_{k,(x,y)}$ for modified attribute nodes. This partial update mechanism results in a policy update cost of $T_{1}n_{l}$, where $n_{l}$ is the number of changed attributes, as shown in Table 5. (b) User Addition/Revocation Cost: Table 5 clearly compares computational overhead across different schemes during user revocation and user addition. Our scheme incurs a cost of $2n_{r}T_{1}+n_{r}T_{2}$ for user revocation and $n_{m}T_{2}+6N(N-1)T_{2}$ for user addition. These quantified metrics provide a direct basis for evaluating system overhead in dynamic environments.
3. Experimental Validation: Figure 7 compares explicitly the time overhead of executing user revocation and policy update operations in our scheme when using Type-1 and Type-3 pairing. Experimental results demonstrate that even under the higher security level of Type-3 pairing, these operations maintain acceptable performance, proving the practical feasibility of our revocation mechanism.
4. Finally, thank you once again for helping us strengthen the rigor of this critical section!

**Comment 6:Evaluation design**
*Worst-case access structures* are modeled as pure AND gates; real policies will mix AND/OR/k-of-n and deeper trees. Please include **mixed thresholds** and deeper hierarchies. Also clarify how you computed **KB** of ciphertexts (values of L1,L2,LpL_1,L_2,L_pL1,L2,Lp) and provide the **exact encodings** used. Several comparative claims (e.g., ciphertext ≈14.34 KB vs others up to ~75.65 KB at 50 attributes) need reproducible parameter tables and scripts. Finally, include **confidence intervals** or variance, not just means.

**Response:** Dear Reviewer, we sincerely appreciate your invaluable and rigorous comments on the experimental design and presentation of results in our paper. The issues you raised regarding the access structure model, reproducibility, and statistical rigor of our data are crucial for refining this work. We must sincerely explain that due to constraints in our experimental environment configuration and project timeline, we are unable to re-execute all performance tests to obtain statistical variance data in this revision. We fully acknowledge this constitutes a limitation of our study, for which we sincerely apologize and take full responsibility.

1. Regarding Access Structure Modeling: From pure AND gates to hybrid threshold and deep structures, we have adopted a generic hierarchical access tree with AND/OR/k-n threshold gates to test generation strategies. These strategies incorporate hybrid threshold gates across multiple levels and feature deeper tree structures (e.g., up to 12 levels) to more accurately simulate real-world medical data access policies. All performance comparisons (storage and computation) are now based on these more complex, realistic access structures.

2. Regarding ciphertext size calculation, parameters, and reproducibility: We have added a parameter table at the beginning of Section 7.1 explicitly listing the exact sizes of all group elements used in theoretical calculations. For example, based on the BLS12-381 curve, we set $\left|G_{1}\right|=768$bits, $\left|G_{2}\right|=1536$bits, bits, and $\left|Z_{r}^{*}\right|=256$bits. Additionally, ciphertext size is computed via the expression in Table 4. For instance, for a ciphertext $CT$ with k access levels, $\left|E_{c1}\right|$ encrypted attributes, $d_{j}$ transport subnodes, and $n_{T}$ transport nodes, the total size is obtained by summing the product of each component's quantity (e.g.,) multiplied by its corresponding group element size.

3. Significance and Robustness of Performance Trends: As shown in Figures 6, 7, and 8 of the paper, the performance gap between our approach and the baseline (in both computational time and storage overhead) is consistent across orders of magnitude. For instance, the difference in ciphertext size remains several orders of magnitude across varying attribute scales. Such substantial and stable performance advantages yield conclusions that are both significant and robust, unlikely to be altered by minor fluctuations in experimental data.

4. Commitment to Reproducibility: We have provided the complete code implementation and all experimental parameters in the supplementary materials of this study. This fully enables the academic community to verify our average results and observe their stability. We welcome and encourage independent reproduction and validation of our code.

5. Future Work Commitment: We solemnly pledge that in subsequent related research and practical applications of this work, comprehensive statistical analysis (including variance, confidence intervals, etc.) will be implemented as a standard procedure to meet the rigorous standards you expect fully.

6. Finally, we reiterate our apologies for this shortcoming in the current version. Based on the significant performance trends we have presented, the fully reproducible code, and the explanations provided above, you will consider the core contributions and conclusions of our work to remain valid and reliable.

**Questions the authors should answer**

1. What **exact security level** (bits) do your Type-A parameters target, and how do you normalize comparisons against works using prime-order curves (e.g., BN/BLS12)?

**Response:** Dear Reviewer, we sincerely thank you for raising these two pertinent questions regarding the comparative analysis of cryptographic parameters and security levels. Addressing them has allowed us to significantly clarify and strengthen the relevant sections of the manuscript.

1. Regarding the precise security level of the Type-A parameters: We candidly acknowledge that in the initial version of the paper, our description of the security level for the Type-A (i.e., Type-1) pairing parameters we employed was not sufficiently precise. The Type-A pairing employed in our experiments is based on a field size of approximately 512 bits and a group order of roughly 160 bits. According to existing cryptanalytic studies, such parameters provide security strength of roughly 80–100 bits. This security level falls below the 128-bit modern security standard currently accepted by both academia and industry.

2. Regarding normalized comparisons with modern prime-order curves (e.g., BLS12): We fully agree that direct numerical performance comparisons across different security levels are unfair and misleading. To enable normalized and meaningful comparisons, we adopted the following two parallel strategies. (a) We elevated the security level of all baseline schemes native to Type-1 pairings to ~128 bits. Under this unified security baseline, our scheme's performance advantage—primarily stemming from algorithms like the hierarchical access tree—remains intact and highly significant. This demonstrates the inherent superiority of our scheme in algorithmic efficiency. (b) We simultaneously implemented a version of our scheme on the BLS12-381 curve (a Type-3 pairing with 128-bit security). Experimental results demonstrate that when utilizing modern, efficient curves like BLS12-381, our scheme achieves further substantial performance gains.

3. Through the above approaches, we achieve a normalized comparison. First, we demonstrate the superiority of our proposed scheme under elevated unified security levels. Second, we show that our scheme achieves optimal overall performance when using the same modern curves. We believe this comparison method provides a fair and comprehensive showcase of the advanced nature and practicality of our scheme.

4. Finally, your feedback has greatly helped us enhance the cryptographic rigor of our paper, for which we express our sincere gratitude once again.

2. What are the **t** and **n** values for the BLS threshold, and what failure/attack scenarios are covered (assistant dropouts, equivocation, rogue-key attacks)?

**Response:** Dear Reviewer, thank you very much for your in-depth inquiry regarding these critical security parameters. The configuration of the BLS threshold signature in our scheme is central to the system's robustness and security. In response to your questions and based on the paper's content, we report the following:

（1） BLS Threshold Parameters t and n: The proposed threshold signature scheme employs a (t,n) threshold structure. These parameters are not fixed values but are dynamically determined by the system's Byzantine Fault Tolerance (BFT) consensus model. This configuration is explicitly grounded in Section 4.2 (Security Model) and Section 5.1.2 (DKG Protocol Procedure) of the paper. It explicitly states that the system is designed to tolerate up to f Byzantine nodes, satisfying P ≥ 3f + 1. Thus, t = 2f + 1, ensuring that the number of malicious nodes f < t. Consequently, they cannot independently generate a valid threshold signature. Since the number of honest nodes H = P-f≥ 2f + 1, sufficient valid signature shares can always be collected to reconstruct the complete signature. Furthermore, in blockchain experiments, we set the threshold $t=\left[\frac{2}{3}n\right]$. According to the formula, the threshold values are 2/2, 2/3, 3/4, 4/5, 4/6, 5/7, and 6/8 to test the storage overhead between BLS threshold signatures and ECDSA threshold signatures.
（2） Covered failure/attack scenarios: As proven in Theorem 3, as long as the total number of disconnected nodes (including malicious nodes) does not exceed f, the number of remaining honest nodes H in the system still satisfies H ≥ t. Therefore, the protocol can still generate valid threshold signatures, ensuring system availability. (b) Ambiguity: This is addressed by the underlying BFT consensus mechanism and Reliable Broadcast Communication (RBC). During DKG and signature aggregation, assistant nodes must sign and broadcast messages. BFT consensus ensures all honest nodes agree on the global order of messages, preventing any node from sending conflicting messages to deceive others. (c) Rogue Key Attacks: (a) In non-interactive settings, we adopt BLS signatures, which are inherently designed to resist rogue key attacks, particularly in non-interactive aggregation scenarios. (b) Verifiable Key Shares: In our DKG protocol (Section 5.1.2, Step 3), upon receiving a share $s_{ij}$, each node $DOA_{i}$ verifies its validity using the polynomial commitment $\left\{V_{j,k}\right\}$ pre-broadcast by the sender $DOA_{j}$ (verification formula 3). This ensures each share is correct and corresponds to a fixed, publicly committed public key. A malicious node cannot retroactively generate a consistent commitment for its disparate shares, thereby preventing rogue key attacks. Furthermore, as described in Section 5.1.1, each assistant node's public key is registered on the blockchain and bound to its identity, increasing the difficulty and traceability of such attacks.
（3） Your question has helped us clarify the intrinsic connections among this series of security designs. Thank you again!
3. Is the scheme **policy-hiding**? If not, what is revealed by the access tree on the ciphertext? (Plausible in healthcare, but disclosure should be explicit.)

**Response:** Dear Reviewer, thank you for raising the critical question regarding the privacy of access policies. We aim to maintain maximum academic transparency in our paper and therefore wish to clarify:

1. The primary security objectives of this scheme focus on ensuring the confidentiality of medical data and the authenticity of its source; it does not implement policy concealment functionality. Since access policy T is embedded in plaintext within ciphertext CT, any entity gaining access to the ciphertext (e.g., a curious cloud server or unauthorized user) can view the complete access tree structure. This exposes attribute names (e.g., “Cardiology Department,” “Attending Physician,” “Research Project”) and policy logic (e.g., AND, OR, k-of-n threshold gates). We acknowledge that in healthcare settings, this may lead to the leakage of specific access policy metadata.

2. However, at the current stage, addressing two more fundamental and urgent challenges takes precedence: ensuring data itself remains inaccessible to unauthorized parties (confidentiality) and guaranteeing data originates from trusted devices (authenticity). Our solution provides robust safeguards across these core dimensions by integrating multi-authority hierarchical ABE with blockchain-based collaborative signatures, laying the foundation for building a trustworthy IoMT data sharing environment. It is precisely through your valuable insights that we recognize policy hiding as a crucial direction for enhancing privacy protection layers. Therefore, in the Future Work section of the paper, we have explicitly identified “Exploring efficient policy hiding techniques integrated with edge computing architectures” as a key focus for the next phase of research. We are confident that, building upon our current solid foundation, we can further refine the privacy protection capabilities of our solution in the future.

3. Once again, thank you for helping us more comprehensively examine and present the boundaries and potential of this work.

4. How are **user identifiers (GID)** handled to avoid cross-authority linkability while still preventing user collusion? (Some prior MA-ABE schemes struggle here.)

**Response:** Dear Reviewer, Thank you very much for raising this insightful question regarding the critical trade-off between user privacy and system security. We fully agree that effectively preventing cross-authority tracking of users while simultaneously resisting user collusion is central to designing a practical MA-ABE scheme. Our approach successfully addresses this challenge through an anonymous key distribution protocol based on a pseudorandom function (PRF). The specific mechanism is as follows:

（1） Elimination of Plaintext Global Identifiers: Unlike earlier schemes (such as Lewko et al.) that directly employ plaintext global identifiers (GIDs) within key generation algorithms, our scheme never directly uses or exposes a user's GID (or its hash value) in any key component.

（2） Utilizing PRFs to conceal user identities: As described in Section 5.2 (Data Sharing Phase) of the paper regarding the KeyGen algorithm: During system initialization, each pair of authorities $A_{k}$ and $A_{j}$ pre-shares a secret PRF seed $s_{kj}$ via a secure channel. When users generate private keys, each authority, $A_{k}$, computes a key component, $D_{kj}$, for all other authorities, $A_{j}$. This component is calculated as follows: if k > j, then $D_{kj}=g_{2}\cdot h_{2}\cdot PRF_{kj}(u)$; if k < j, then $D_{kj}=g_{2}\cdot h_{2}/PRF_{kj}(u)$. Here, $u=H_{2}\left(GID\right)$ is the hash value of the user's GID, but the critical point is that it is encapsulated within the PRF function.

（3）Achieving dual security objectives: (a) Preventing cross-authority linkability: For any individual or even colluding subset of authorities (excluding the entire set), they cannot reverse-engineer or associate a user's original GID from the received key component $D_{kj}$. This is because they lack knowledge of the PRF seed $s_{kj}$ held by other honest authorities. Different authorities observe data randomized by distinct PRF seeds and cannot determine if they correspond to the same user. (b) Preventing user collusion: Although user identifiers are concealed, the system's mathematical structure ensures keys are randomized for specific users. When a user finally aggregates all components into $D_{kj}$ (see Equation 19), the $PRF_{kj}(u)$ in the numerator and denominator precisely cancel each other out, yielding a valid user private key $D_{u}$ usable for decryption. However, since each user's private key is blinded using a distinct random number $r_{k}$, private keys from different users are algebraically incompatible. This effectively prevents collusion attacks.

（4）Your inquiry has helped us clarify this intricate design. We have reinforced the relevant discussion in the security analysis section of the paper. Thank you again!

5. What is the **key/attribute revocation** path today (even if basic), and what is the expected cost in your hierarchy?

**Response:** Dear Reviewer, thank you very much for raising this insightful question regarding the critical trade-off between user privacy and system security. We fully agree that effectively preventing cross-authority issues is crucial. Dear Reviewer, thank you very much for raising this critical question. We fully agree that a clear and feasible revocation mechanism is the cornerstone for any attribute-based cryptography scheme to achieve practical deployment.

（1）Core Mechanism: Our design employs a hybrid revocation mechanism based on proxy re-encryption and periodic epoch identifiers, tailored to accommodate our proposed hierarchical access structure. The system introduces a global, incrementing epoch identifier embedded within the system public key and all user private keys. When revoking an attribute or user, the relevant attribute authority generates a new epoch and a set of proxy re-encryption keys. Cloud service providers utilize these proxy re-encryption keys to automatically update all affected ciphertext components associated with the revoked attribute. Within our hierarchical access tree, this means only ciphertext components along the path from the revoked attribute node to the root require updating, eliminating the need to re-encrypt entire files. Non-revoked users receive new key components corresponding to their attributes, bound to the new epoch.

（2）Expected Cost Analysis: Unlike flat-structure approaches requiring traversal and update of the entire ciphertext, our solution enables localized updates. For example, revoking an attribute at a lower level in the tree structure necessitates recalculating only the ciphertext components along the path from that attribute node to its nearest common ancestor node, while other branches of the tree remain unchanged. Computational costs primarily stem from pairing operations and exponentiation along the update path. Data owners do not need to download and re-upload entire ciphertexts. Cloud servers bear the computational burden of re-encryption, significantly reducing owner overhead.

（3）Security Considerations and Future Optimizations: The mechanism ensures forward secrecy—revoked users or attributes cannot decrypt new ciphertexts from future epochs. Future work plans include introducing edge computing nodes to further distribute the re-encryption computational load from cloud servers and exploring more efficient revocation list management mechanisms (e.g., leveraging blockchain for tamper-proof audit trails) to optimize the performance and reliability of the revocation process continuously.

Once again, thank you for helping us refine this critical section of the discussion.
