# Supplementary material for: A blockchain-based multi-authority hierarchical attribute encrypted data sharing scheme in the Internet of Medical Things
Source: PLoS One. 2026 May 27;21(5):e0349767. doi: 10.1371/journal.pone.0349767 (PMC13215620; doi:10.1371/journal.pone.0349767)
Supplement: Attachment — Submitted filename: Response to Reviewers..docx [file pone.0349767.s007.docx]

Dear Editors and Reviewers:

Thank you for giving us the opportunity to revise our manuscript. We sincerely appreciate the time and effort you have dedicated to providing insightful feedback on our work. We have carefully considered all comments and suggestions and have made revisions to the manuscript accordingly.Our point-by-point responses to the comments are listed below.

**Responses to comments from Reviewer#1**

**Comment 1:**The manuscript should address several issues related to clarity, presentation, rigor of evaluation, and positioning in relation to existing literature.
**Response:** Thank you for your valuable suggestions. We have systematically revised the Introduction (Section 1) and Related Work (Section 2) to establish a clear logical framework and define the positioning of this study. The revisions follow a “Problem-Literature-Gap-Contribution” structure.
1.1 Revision of Introduction (Section 1)
Three core challenges are explicitly stated: In the second paragraph of Section 1, we clearly articulate three technical bottlenecks faced by existing CP-ABE schemes in IoMT scenarios:
Challenge 1: Data sources lack lightweight mechanisms for authenticity verification.
Challenge 2: Traditional CP-ABE schemes struggle to adapt to the hierarchical structure of medical data.
Challenge 3: The trust model based on a single centralized authorization faces single-point-of-failure risks and user revocation issues.

Mapping Contributions to Challenges: In Section 1, Paragraph 4, we explicitly map our four contributions to the three challenges:
Challenge 1 → Contribution 1: Collaborative authentication mechanism integrating DKG and BLS threshold signatures.
Challenge 2 → Contribution 2: Multi-authority CP-ABE mechanism based on hierarchical access trees.
Challenge 3 → Contributions 3 and 4: Periodic revocation mechanism based on proxy re-encryption and security analysis.
Technical Selection Rationale: We also clarified the rationale for choosing Type-3 pairing curves (BLS12-381) over traditional Type-1 pairings: Type-3 curves offer shorter ciphertexts and higher computational efficiency at equivalent security levels, directly addressing the efficiency demands of resource-constrained IoMT devices.
1.2 Revision of Related Work (Section 2)
We restructured Section 2 into three subsections directly corresponding to the three challenges outlined in the introduction:
Multi-authority CP-ABE and its revocation mechanism → Corresponding to Challenge 3.
Decentralized and hierarchical CP-ABE → Corresponding to Challenge 2.
Source authentication and blockchain-assisted auditing → Corresponding to Challenge 1.
Defining Research Gaps: At the end of each subsection, we clearly identify limitations in existing work:
End of Section 2, Paragraph 1: “...significant communication overhead severely limits scalability when handling large numbers of endpoints.” → Addressed by Contribution 3 (low-overhead revocation mechanism).
End of Section 2, Paragraph 2: “...most hierarchical schemes focus solely on post-storage static confidentiality, neglecting verification of data source authenticity at the perception layer.” → Addressed by Contribution 1 (collaborative authentication mechanism).
End of Section 2, Paragraph 3: “...overreliance on single-device credentials compromises distributed fault tolerance...forcing terminals to perform energy-intensive traditional bilinear pairing operations.” → Addressed by Type-3 curves and distributed trust architecture.
1.3 Functional Comparison in Table 1 and Positioning of Type-3
In the newly added Table 1 at the end of Section 2, we clearly demonstrate, through comparison, that all schemes using Type-1 curves fail to simultaneously fulfill all functional requirements of IoMT scenarios, whereas our scheme, employing Type-3 curves, achieves the first-ever integration of all functionalities.

We once again extend our gratitude to the reviewers and editors for their valuable time and constructive feedback. We believe the revised manuscript has been significantly improved and hope it now meets your journal's publication standards.
Should you require further clarification, please do not hesitate to contact us.
