# Supplementary material for: A blockchain-based multi-authority hierarchical attribute encrypted data sharing scheme in the Internet of Medical Things
Source: PLoS One. 2026 May 27;21(5):e0349767. doi: 10.1371/journal.pone.0349767 (PMC13215620; doi:10.1371/journal.pone.0349767)
Supplement: Attachment — Submitted filename: Response to Reviewer.docx [file pone.0349767.s008.docx]

**Dear Editors and Reviewers:**

Thank you for allowing us to revise our manuscript. We sincerely appreciate the time and effort you have devoted to our work, as well as the insightful comments you have provided. We have carefully considered all comments and suggestions and have made the corresponding revisions to the manuscript. All revisions are highlighted in yellow in the revised version. Our point-by-point responses to each comment are as follows:

**Responses to comments from Reviewer#1**

**Comment 1: Clarity and Presentation**

（1）**The notation is dense and sometimes inconsistently formatted. A consolidated notation table including all symbols (with uniform indexing) would improve readability.**

**Response:** We are very grateful for the reviewers' constructive suggestions. The DKG protocol and the hierarchical multi-authority CP-ABE scheme discussed in this paper involve numerous parameters and indices, which can indeed make the text difficult for readers to follow. To address this issue and improve the clarity of the manuscript, we have made the following improvements:

(i) At the beginning of Section 5 on the BMHADS scheme in the revised manuscript, we have revised Table 1 to provide a comprehensive summary of all core symbols used in the paper, including their physical meanings and unified index definitions.
(ii) We have carefully reviewed the entire text to verify the usage of each symbol in the table, ensuring that the meanings of all symbols in the main text align with the definitions in the table and that their formatting is consistent. These improvements significantly enhance the clarity and reproducibility of the paper.
（2）**Some equations lack intuitive explanations. In particular, the derivation logic in the decryption phase and hierarchical node computation would benefit from a short narrative explanation alongside the formulas.**

**Response:** We thank the reviewers for their suggestions. We have optimized and streamlined the formulas in the decryption phase, removing unnecessary redundant expressions to highlight the core derivation logic. Additionally, we have added brief explanatory notes next to (or immediately following) each key formula to clarify the steps of algebraic elimination and the meaning of the hierarchical node recursive computation. For example:

（i）The core logic of Equation (28) is de-blinding. To ensure security, a disturbance term based on $h_{2}$ is introduced into the ciphertext component. During decryption, the Decryptor (DU) performs pairing operations on the ciphertext using its private key $D_{U}$ and aggregates the attribute secrets by recursively executing the DecNode function. The fractional operation in Equation (28) is ingeniously designed to precisely offset all blinding factors related to $h_{2}$, thereby extracting a pure bilinear pairing value $F_{m}$ that contains only the system’s core secret $\delta_{k}$ and the hierarchical random quantity.
（ii）Equation (29) illustrates the key-passing mechanism for hierarchical access control. The ciphertext component $\bar{C}_{(x_{m},y_{m}),j'}$ acts as an encryption bridge between parent and child nodes. By hashing the parent node’s decrypted $F_{m}$ and using it as a divisor, the DU can unlock the child node’s secret from $\bar{C}_{(x_{m},y_{m}),j'}$. Algebraically, the step leverages the polynomial $q\left(0\right)$ to ensure that only users who successfully decrypted the parent node’s level can recursively derive the child node’s intermediate values, achieving ordered downward propagation of access permissions in the hierarchy.
（iii）Equation (30) represents the final extraction step. The ciphertext component $\tilde{C}_{m}$ is essentially the result of masking the content key $ck_{m}$ with the intermediate pairing value $F_{m}$. Once $F_{m}$ is successfully reconstructed, simple algebraic elimination (division in group operations) can be used to remove the mask, thereby accurately restoring the original content key $ck_{m}$. Finally, the message $M$ is recovered using.
（3）**Figures (e.g., system model and DKG process diagrams) are helpful but should include clearer legends and step-number alignment with the textual description.**

**Response:** We thank the reviewers for their detailed suggestions. We have made the following improvements to the system model diagram in Figure 3, the DKG protocol flowchart in Figure 4, and the data sharing flowchart in Figure 5:

（i）First, regarding the system model diagram in Figure 3, we have drawn it strictly in accordance with the proposed BMHADS scheme. Since the DKG protocol in Section 5.1 comprises seven phases—registration, initialization, commitment and share distribution, complaint, key reconstruction, signing, and key update—we have labeled the protocol as a whole in Figure 3 as the “DKG Protocol” phase. We have explicitly noted in the figure caption that the specific phases and detailed interaction processes are described in Figure 4.
（ii）Second, for the DKG protocol flowchart in Figure 4, we strictly adhered to the DKG protocol scheme described in Section 5.1 and labeled the seven phases—registration, initialization, commitment and share distribution, complaint, key reconstruction, signing, and key update—using circled numbers (①、②、...⑦) in the figure. This numbering ensures consistency with the main text stage divisions and helps readers compare and understand the protocol flow.
（iii）Finally, regarding the data sharing stage, we have divided this stage into seven sub-sections, labeled in the figure as: 1. System Setup, 2. Authority Setup, 3. Key Generation, 4. Encryption, 5. Verification, 6. Decryption, and 7. Revocation. The labels in the diagram correspond one-to-one with the seven sections mentioned above. Additionally, the formal algorithm names for each subsection are listed at the beginning of the label, specifically: System Setup（CA.Setup）、Authority Setup（Auth.Setup）、Key Generation (KeyGen), Encryption (DO.Enc), Verification (DU.Verify)、 Decryption (DU.Dec) and Revocation（Revocation）。
（4）**Minor grammatical issues and awkward phrasing remain throughout the manuscript and should be addressed through careful language editing.**

**Response:** We want to thank the reviewers for their detailed feedback. We have systematically revised the entire manuscript, as outlined below:

（i）First, we ran the manuscript through multiple rounds of scanning using a professional grammar-checking tool (Grammarly) to correct grammatical issues such as subject-verb agreement, incorrect article usage, and tense inconsistencies.
（ii）Second, we manually reviewed and rewrote each instance of “awkward phrasing” and redundant expressions flagged by the tool. For example, we removed unnecessary “this” and “that,” simplified complex sentences, and standardized the use of key terminology.
（iii）We believe that this careful linguistic editing has improved the manuscript’s grammatical quality and fluency. We want to thank the reviewers once again for their valuable feedback.

**Comment 2:Technical Soundness and Rigor**

（1）**The selective security model is adopted under the DBDH assumption. Please clarify whether full security (adaptive security) could be achieved or discuss limitations of selective security in practical deployment.**

**Response:** We are very grateful to the reviewers for their valuable feedback. We have demonstrated the selective security of the proposed BMHADS scheme under the DBDH difficulty assumption. In response to your suggestion, we have added Section 4.4 to discuss the security model.

（i）We explained that while total security provides stronger guarantees, this model typically requires the introduction of dual-system encryption techniques, which would result in an exponential increase in ciphertext length and a surge in computational overhead, making it unsuitable for the resource-constrained nature of IoMT devices.
（ii）The main limitation of the selective security model is that it requires the adversary to submit the target access structure for attack before system initialization. This limits, to some extent, the simulation of an adversary’s ability to dynamically adjust attack strategies. However, in real-world distributed healthcare scenarios, user attributes and permissions (e.g., attending physicians, researchers, head nurses) and system access policies are typically determined by organizational structures and are relatively stable, changing infrequently within milliseconds. Therefore, selective security is sufficient to cover the threat models in the vast majority of real-world applications.
（2）**The security proof outline should be expanded. Currently, the manuscript states provable security but does not provide sufficient reduction details to fully assess rigor.**

**Response:** We are very grateful for the reviewers’ valuable feedback. We fully agree with your assessment that a thorough security reduction process is central to evaluating the rigor of our approach. In response to your suggestions, we have significantly expanded and refined the “Security Analysis” section (Section 6) of the original manuscript. The specific revisions are as follows:

（i）Detailed reduction process: We have added the complete interaction details between Simulator and Adversary. We clearly demonstrate how utilizes mathematical problems (such as DBDH instances) to set parameters and embed them into the public component.
（ii）Refined simulation phases: Following the standard security game model, we have derived in detail every algebraic step for the Setup phase, Private Key Query Phase 1, Challenge phase, and Query Phase 2. In particular, for the private key query, we detail how the simulator constructs a response key with a valid distribution using algebraic cancellation techniques without knowing the core secret (e.g., ab).
（iii）We provide a calculation of the advantage that Simulator gains from solving the puzzle and conduct a detailed analysis of the probability that the simulation will not terminate prematurely.
（iv）Consistency Proof: We have included algebraic derivations such as polynomial expansions (e.g., Equation 32) to demonstrate that the ciphertext and keys generated by the simulation are mathematically identical to those of the genuine scheme.
（3）**The resistance to Sybil attacks relies on economic staking and smart contracts. Please clarify the threat model assumptions regarding blockchain trust and the adversary’s financial capacity.**

**Response:** We thank the reviewers for their insightful comments. In distributed key generation (DKG) protocols, resistance to Sybil attacks is a prerequisite for ensuring system robustness. In Section 4.2 (Threat Model and Assumptions) of the revised draft, we have further refined and clarified the relevant assumptions:

（i）Blockchain Trust Assumption: We assume that the underlying consortium blockchain employs a Byzantine Fault Tolerance (BFT) consensus mechanism. This mechanism ensures that the global state tree (containing all nodes’ registered addresses, public keys, staking amounts, penalty records, etc.) remains tamper-proof and fully consistent across all honest nodes; no adversary can forge historical data or identity records by tampering with a local ledger. At the same time, smart contracts are treated as “honest arbiters,” and their execution logic (including identity activation, staking custody, and slashing rules) is strictly enforced by the decentralized consensus protocol. Adversaries cannot bypass the contract logic to activate unauthorized identities unless they meet the necessary conditions (such as failing to submit valid staking or complete identity verification).
（ii）We assume that adversaries are financially rational, meaning their attack objective is to maximize illicit gains (such as stealing medical data), and they will only launch an attack when the expected gains exceed the total cost of the attack (including hardware costs, staking losses, etc.). To prevent Sybil attacks (where an adversary creates multiple fake identities to gain control of the DKG protocol), the system enforces a minimum staking threshold $E'_{\min}=\left(f+1\right)\cdot\Omega$ during the node registration phase, where $f$ is the maximum number of Byzantine nodes the system can tolerate, and $\Omega$ is the preset minimum staking amount per node. By setting $\Omega$, $E'_{\min}$ is made significantly higher than the potential illicit gains from stealing medical data, thereby rendering large-scale Sybil attacks economically unfeasible. Furthermore, if any node in the DKG protocol sends inconsistent commitments or invalid shares, honest nodes will initiate a complaint via BFT consensus. The smart contract will automatically confiscate all of that node’s staked funds and remove it from the pool of eligible nodes, further increasing the cost of an attack.
（4）**In the DKG protocol, the communication complexity and on-chain overhead should be formally analyzed.**

**Response:** We thank the reviewers for their valuable suggestions. We fully agree that formalized cost analysis is crucial for evaluating the scalability of distributed protocols. In the revised version, we have added Section 7.1.4 to discuss the communication complexity and on-chain storage overhead of the DKG protocol. The specific modifications are as follows:

（i）**Formal derivation of communication complexity:** We have modeled the DKG protocol layer by layer, covering registration, commitment broadcasting, share distribution, the consensus phase, and the key update phase. Our derivation shows that the protocol's total communication complexity is $O\left(P^{2}\right)$. Although $O\left(P^{2}\right)$ theoretically grows quadratically with the number of nodes $P$, our experiments, as shown in Figure 8(c), confirm that the measured consensus latency is only 280 ms at $P=100$. Consequently, with the adoption of the BFT mechanism, this complexity order is entirely acceptable in real-world IoMT network environments.
（ii）Formal Derivation of On-Chain Storage Overhead: We established a cumulative model of total on-chain storage requirements, covering node registration information, commitment matrices, hash evidence, and aggregated signatures. The derivation shows that the storage complexity of DKG state updates is $O\left(Pt\right)$, while the space complexity of authentication during the collaboration phase is $O\left(1\right)$. Formal calculations show that under a high-load configuration with $P=100$, the on-chain data increment generated by a single "period switch" is approximately 1.27 MB. Since this payload is far below the block capacity limit, and the collaborative signature exhibits constant-scale characteristics, it will not affect the scalability of IoMT devices as the number of nodes increases.

**Comment 3:Performance Evaluation**

（1）**Provide clearer baseline comparisons and justify why selected schemes represent state-of-the-art.**

**Response:** We thank the reviewers for their comments. In response to the reviewer's suggestion to "provide a clearer baseline comparison," we have restructured Sections 7.1 (Theoretical Analysis) and 7.2 (Experimental Analysis) to ensure the rigor and clarity of the comparisons:

（i） Established a classification framework for comparison: We no longer conduct indiscriminate mixed comparisons but instead categorize the baseline schemes into three groups based on their functional evolution logic: the Functional Foundation Group ([15–18]), the Architectural Integration Group ([21]), and the Cutting-Edge Blockchain Group ([32, 33]). This classification method more clearly demonstrates the improvements of our scheme across different technical dimensions.
（ii） Implemented parameter normalization: In previous comparisons, the descriptions of security strength for each scheme were vague. In the revised version, we explicitly state that all schemes are compared at the 128-bit security level (Level 1). As shown in Table 8, we ensured the comparability of technical metrics by adjusting the base field sizes of the Type-1 and Type-3 curves.
（2）**Include explicit parameter settings (curve type, security level, hardware configuration).**

**Response:** We sincerely thank the reviewers for their suggestions. To ensure the transparency and rigor of our technical benchmarks, we have added Table 7 to Section 7.2 of the revised manuscript. This table provides a detailed list of the hardware environment, software libraries, and specific cryptographic parameters used in the experiments.

1. Clear Parameter Settings: As shown in Table 7, we have clearly defined the Type-1 hyperelliptic curve used in the comparison scheme and the Type-3 BLS12-381 curve used in our scheme. It is worth noting that, to ensure fairness in the comparison, both curves were configured to meet the 128-bit security level. Additionally, the table details the hardware (Intel i7-9750H) and software (MIRACL library) configurations to ensure the reproducibility of the experiments.
2. Quantitative Proof of Technological Advancement: Table 7 provides quantitative evidence explaining why the selection of Type-3 asymmetric pairs represents the state-of-the-art technology in the IoMT field.
（i）Modularity Efficiency: To achieve a 128-bit security level, the Type-1 curve requires a base field modulus $q$ of 1,536 bits, whereas the Type-3 curve requires only 381 bits. This significantly reduces the computational burden on resource-constrained IoMT sensors.
（ii）Storage optimization: The size of group elements is reduced from 3,072 bits in Type-1 to 768 bits in Type-3. This substantial size reduction directly translates to shorter ciphertexts and lower communication overhead.
（iii）Curve Rationality: The BLS12-381 curve is currently recognized in both academia and industry as a highly efficient paired curve. Compared to the Type-1 curve used in traditional schemes, it achieves a better balance between security and performance.
（3）**Report scalability analysis (e.g., increasing number of authorities, attributes, or hierarchy depth).**

**Response:** We thank the reviewers for their suggestions. We have increased the number of authorities from 3 to 10, with the following specific modifications:

（i）In the experimental analysis section, we adjusted the number of authorities from $N=3$ to $N=10$ and redrew all relevant experimental figures. The experimental results show that key generation time, user decryption time, and other key metrics increase linearly with the number of authorities. Compared to the $N=3$ setting, under the $N=10$ configuration, when the number of attributes is less than 20, the key generation time of the proposed BMHADS scheme is slightly higher than that of scheme [10]; when the number of attributes is less than 13, it is slightly higher than that of schemes [10, 11]. However, when the number of attributes exceeds 20, the key generation time of our scheme is consistently lower than that of schemes [10, 11, 12]. During the decryption phase, whether for files with 4 to 12 levels or for scenarios with a fixed number of 5 files and attribute counts ranging from 10 to 50, the decryption time of this scheme consistently outperforms that of schemes [27, 26, 16, 15, 13]. Compared to the results for $N=3$, the decryption time of this scheme is slightly lower than that of schemes [26, 16] when N=10. Furthermore, during the encryption phase, this scheme outperforms [27, 26, 16, 15, 13] regardless of whether the number of files or attributes is increased.
（ii）Regarding user revocation, we omitted the comparison with Type-1 pairings because both the power operation time and the bilinear pairing time of Type-3 pairings are superior to those of Type-1. We compared the user enrollment overhead with Scheme [12]. Since the number of authorities is set to 10 and our scheme requires executing an anonymous exchange protocol during key generation, Scheme [12] has a shorter key generation time than ours when the number of attributes is less than 60. However, the key generation time of our scheme increases more slowly as the number of attributes grows; when the number of attributes exceeds 60, our scheme’s key generation time is shorter than that of Scheme [12].
（4）**Separate computation time, communication overhead, and blockchain interaction costs in the performance tables.**

**Response:** We thank the reviewers for their suggestions. We fully agree that this multidimensional classification and evaluation are crucial for clearly identifying performance bottlenecks in distributed architectures. In the blockchain experiments of the revised manuscript, we have reclassified the total overhead of the DKG protocol. We no longer report total elapsed time but instead break down the cost precisely into the following three distinct dimensions:

（i）Computational time: includes the time taken for parallel DKG share refreshes on the assistant node side (81.08 ms) and the time taken for threshold signature aggregation during collaborative authentication (18.51 ms), which demonstrates the system's exceptional performance in terms of CPU resource consumption.
（ii）Communication overhead: We quantified the actual message load at the network layer, including DKG commitment broadcasts (1.23 MB) and peer-to-peer share distribution between nodes (2.14 MB).
（iii）Blockchain interaction costs: We calculated blockchain-specific cost metrics separately, including the on-chain storage increment generated by a single epoch transition (1.27 MB) and the consensus confirmation time under a high-load scenario of 300 TPS (280 ms).
（iv）Through the quantification in the above categories, we further demonstrate the system's real-time guarantees: specifically, that high-frequency data-plane encryption (11.50 ms–33.5 ms) and low-frequency control-plane consensus (280 ms) are decoupled in terms of their paths, and that consensus latency does not block the availability of core medical data. The relevant modifications are reflected in Table 9 and the subsequent analysis sections.
（5）**Discuss practical deployment feasibility in real IoMT environments with constrained devices.**

**Response:** We thank the reviewers for their suggestions. We have thoroughly demonstrated the practical feasibility of deploying this scheme on resource-constrained IoMT devices through our experiments (Figures 8 and 9), as evidenced by the following:

（i）Computational feasibility: End devices need to perform a single scalar multiplication for the BLS signature; as shown in Figure 8(b), the aggregated signature is always 96 bytes. Furthermore, data encryption is handled by the DO; in practical tests, the time required for layered encryption ranged from 11.71 to 33.5 ms when the number of attributes ranged from 10 to 50. The majority of computationally intensive tasks—including BFT consensus, DKG protocol execution, and bilinear pairing operations—are offloaded to auxiliary nodes with sufficient computing power. Resource-constrained IoMT endpoints are responsible only for lightweight signing and basic communication tasks, thereby effectively extending battery life and reducing thermal dissipation requirements.
（ii）Low Storage Footprint: As shown in Figure 8(a), the on-chain storage increment is only 1.27 MB at 100 nodes, and the terminal needs to store only its own private key and the system public key, totaling 224 bytes, which meets the storage constraints of typical low-power MCUs.
（iii）Real-time Performance Guarantee: As shown in Figure 8(c), the consensus latency is 280 ms, and the control plane is decoupled from the data plane. High-frequency physiological data transmitted off-chain requires 11.71–33.5 ms, ensuring that consensus processes do not block real-time monitoring streams. Even under an extreme attack scenario where 30% of nodes are malicious, the latency remains at 250 ms, which is below the clinical real-time response threshold (1 second).

In summary, this solution is adapted to constrained IoMT hardware in terms of computation, storage, and communication, and is feasible for deployment in real-world environments. Related discussions have been added at the end of Section 7.2.

**Comment 4: Positioning with Respect to Existing Literature**

（1）**A more critical comparison of how this scheme differs mathematically from existing hierarchical MA-ABE constructions.**

**Response:** We sincerely thank the reviewers for this insightful suggestion. We have elaborated on the relevant prior work (Section 2) and prerequisite knowledge (Section 3.1) to clarify the fundamental differences between the Type-3 asymmetric construction adopted in this study and existing Type-1 symmetric schemes (e.g., [20,21]). The key mathematical structural differences are as follows:

（i）Transformation of algebraic structures: Existing multi-authority hierarchical CP-ABE schemes (such as those in [20,21]) are primarily based on Type-1 symmetric pairings (i.e.,), where computable homomorphisms exist between the groups. Although this symmetry simplifies the mapping logic from attributes to group elements, it introduces significant security risks at high security levels. In contrast, the proposed BMHADS scheme employs a Type-3 asymmetric pairing (i.e.,), and no such mapping exists between the groups. This isomorphic structure effectively eliminates the security-degradation risks introduced by group homomorphisms, providing a more robust mathematical foundation for the Discrete Logarithm Problem (DLP).
（ii）The "parameter inflation problem" in hierarchical access trees: From a numerical computation perspective, to achieve a 128-bit security level and resist MOV attacks, Type-1 curves require a finite field bit width of approximately 3,072 bits. In a hierarchical access tree, this results in linear, exponential growth in ciphertext size. In contrast, our Type-3 construction (using the BLS12-381 curve) requires only 381 group elements to achieve equivalent security. From a mathematical perspective, this fundamentally resolves the inherent redundancy in the algebraic representation of the access tree, enabling the scheme to operate more efficiently on resource-constrained IoMT endpoints.
（iii）Design trade-offs between complexity and efficiency: We have directly addressed the trade-offs regarding implementation complexity. In Type-1 schemes, parameter mapping is uniform and simple. In contrast, the Type-3 structure requires implementing a complex parameter partitioning strategy that precisely allocates secret shares and attribute components to heterogeneous groups. Although this increases the algebraic complexity of the system design, this evolution is a necessary mathematical choice to achieve the lightweight characteristics required for modern medical IoT environments.
（iv）We believe that through these revisions, we have provided the "critical comparison" requested by the reviewers and clearly demonstrated the mathematical structural superiority of this work over previous hierarchical MA-ABE schemes.
（2）**Clear discussion of trade-offs introduced by using Type-3 pairings versus Type-1 pairings beyond efficiency (e.g., implementation complexity).**

**Response:** We are very grateful to the reviewer for this insightful suggestion. In the revised manuscript (particularly in Section 3.1 and the related work in Chapter 2), we have added an in-depth trade-off analysis of Type-3 and Type-1 pairings across mathematical structure, security, and implementation complexity. We emphasize that the choice of Type-3 pairings is not merely driven by computational efficiency, but rather a comprehensive decision that balances system robustness and the complexity of algebraic design. The specific trade-offs are as follows:

(i) Significant increase in implementation complexity: For Type-1 symmetric pairings, since and an efficient homomorphic mapping exists, the parameter mapping logic is highly intuitive, allowing developers to map all attribute indices to the same group. For Type-3 asymmetric pairings, because, and no valid mapping exists between the groups, our approach must implement a rigorous parameter partitioning strategy, requiring meticulous determination during the system design phase of which secret shares, public parameters, and attribute components should be assigned to the space and which to the  space. Such cross-group coordination significantly increases both the difficulty of algorithm design and the complexity of engineering implementation.
(ii) **Security robustness and group homomorphism risks:** Although Type-1 is simple to implement, the high symmetry between groups makes it vulnerable to MOV attacks and security degradation threats based on group homomorphism. To mitigate these risks and achieve 128-bit security, Type-1 must sacrifice storage space (requiring a bit width of 3072 bits). In contrast, by eliminating unnecessary mapping relationships between groups, Type-3 cuts off attack paths that exploit group isomorphism at the underlying mathematical level, thereby providing a purer and more robust security guarantee for the discrete logarithm problem (DLP), such as ECDLP and FFDLP.
(iii) Trade-off between hash algorithms and algebraic properties: Type-1 supports simple hash-to-curve operations, with a more straightforward algorithmic workflow. Type-3, however, requires the use of more complex irreversible hash functions and must ensure consistent operational logic across non-isomorphic groups.
(iv) We explicitly state in our paper that adopting a Type-3 construction involves a trade-off between complexity, security, and lightweight implementation. Although increased implementation complexity places greater demands on system development, it fundamentally resolves the intractable issues of "parameter bloat" and "security decay" inherent in symmetric pairing schemes. Such a resolution is crucial for Internet of Medical Things (IoMT) scenarios, where resources are severely constrained, and security requirements are extremely high.
（3）**Clarification on how the proposed collaborative authentication mechanism substantially improves over existing aggregate signature approaches.**

**Response:** We are very grateful to the reviewers for this important question. In the revised manuscript, we have provided a thorough clarification of the differences between our collaborative authentication mechanism and traditional aggregated signature methods (e.g., [32, 33]). We emphasize that our scheme is not merely a simple signature compression but rather achieves a paradigm shift from "individual credibility" to "collective consensus." The specific improvements are reflected in the following three aspects:

（i）Transition from a "collect-then-compress" logic to a threshold consensus logic: Existing aggregated signature methods (e.g., [32, 33]) typically follow a "collect-then-compress" logic, which essentially involves the simple superposition and mathematical compression of multiple independent signatures. Such an approach means that while a verifier can validate multiple sources at once, each signature still relies entirely on the single credential of the corresponding device. In contrast, the proposed scheme integrates a distributed key generation (DKG) protocol to construct a $\left(t,P\right)$ threshold signature mechanism. A single device no longer generates signatures; instead, at least $t$ assistant nodes must collaborate and reach consensus. Consequently, the verification of data source authenticity evolves from "single-point trust" to "decentralized consensus."
（ii）Introduction of Decentralized Fault Tolerance: In an aggregated signature model, if a hacker hijacks a specific device and obtains its private key credentials, they can easily forge the device's data signature, exposing the system to severe single-point-of-failure risks. In contrast, the collaborative authentication mechanism in this solution offers exceptional fault tolerance: since private keys are distributed and stored in fragments, no single node possesses the complete credentials. Even if as many as $t-1$ auxiliary nodes are compromised or exhibit malicious behavior, an attacker remains unable to forge a valid signature. This mathematical hard constraint ensures the absolute authenticity of data flows in IoMT environments—a capability lacking in traditional aggregated signature schemes.
（iii）Enhanced proactive defense capabilities at the perception layer: Aggregated signatures are typically a passive space-optimization technique focused on reducing storage load. In contrast, our collaborative authentication mechanism serves as a proactive security defense measure. By introducing distributed verification via assistant nodes at the perception layer, this scheme can intercept fraudulent data using threshold consensus before it enters the blockchain or the cloud. This mechanism shifts the security boundary forward, effectively addressing the defensive vulnerabilities caused by over-reliance on single-device credentials, as discussed in [32, 33].
（iv）We further elaborate on these significant improvements in the sections on related work and system models. By combining DKG with threshold signatures, our approach not only optimizes efficiency but also shifts security depth from "aggregation of individual signatures" to "distributed consensus authentication."

**Comment 5: Practical Considerations**

（1）**The manuscript should discuss how key escrow issues are mitigated in the multi-authority setting.**

**Response:** Dear Reviewer, thank you very much for this critical suggestion. In the revised manuscript, we have updated Section 6, "Analysis of Authority Collusion and Key Escrow Mitigations," to include a formal discussion of this issue. The proposed BMHADS scheme effectively mitigates key escrow risks through its decentralized key construction logic; the user's global private key is the product of all N independent authority components (as shown in Equation 19). We clarify the following points:

（i）No single authority (including even a central CA) possesses sufficient information to derive a valid, complete private key independently.
（ii）Our security proof (Theorem 1) establishes that the system remains secure even if up to $N-2$ authorities are compromised, ensuring that at least two honest authorities protect the master secret.
（iii）In practice, even if $N-1$ authorities collude, the master secret cannot be reconstructed, thereby eliminating the risk of a single point of key custody.
（2）**Revocation latency and synchronization in real-world healthcare systems should be examined.**

**Response:** We appreciate the reviewers' suggestions. In the proposed BMHADS scheme, ensuring that all assistant nodes across the network can synchronously and securely update their private key shares is central to guaranteeing the system's business continuity. To address this concern, we have made the following improvements in the revised version:

（i）Clarification of evaluation metrics: We focused our analysis on the delay in key updates taking effect. This metric quantifies the entire process from the moment the system initiates the period-switching command until all 100 assistant nodes complete peer-to-peer (P2P) incremental share exchange, ultimately reaching BFT consensus on the blockchain and finalizing the recording of the new commitment value.
（ii）Quantifying synchronization performance: Experiments were conducted in a large-scale network environment with 100 assistant nodes. Results show that the end-to-end delay for synchronizing key shares across all 100 assistant nodes was only 1.52 seconds.
（iii）Analysis of the Synchronization Mechanism: The 1.52-second latency demonstrates the system's ability to synchronize security states among assistant nodes across medical institutions rapidly. Thanks to the instant finality of BFT consensus, the system ensures that all nodes simultaneously cross the epoch boundary and enter the next security epoch. This robust synchronization mechanism prevents authentication failures due to inconsistent node versions in distributed environments, validating the solution's feasibility for real-world medical collaboration settings.
（3）**The impact of blockchain consensus delay on real-time medical data availability should be analyzed.**

**Response:** We thank the reviewers for their insightful comments. In an IoMT environment, real-time data availability is critical for clinical decision-making. In Section 7.2.3 of the revised manuscript, we provide an in-depth analysis of the impact of consensus latency from two perspectives: architectural design and latency quantification.

（i）Decoupling at the architectural level: The core design principle of this approach is the separation of the control plane and the data plane. In the data plane, high-frequency physiological data collected by IoMT devices is encrypted using a local encryption algorithm (11.50 ms–33.5 ms) and then transmitted directly to the cloud. This path completely bypasses the blockchain consensus process. Therefore, the availability of real-time data uploads and authorized reads depends solely on local computational overhead and network bandwidth, and is unaffected by fluctuations in blockchain performance. From the control plane perspective, the blockchain is responsible only for managing operations such as low-frequency key state updates, node registration, and revocation. The experimentally measured 280 ms consensus latency applies solely to these control commands.
（ii）Quantitative Impact Analysis: In clinical medicine, a latency of less than 1 second is generally considered real-time for remote monitoring. The consensus latency of this solution (280 ms) is far below this threshold. More importantly, due to the adoption of the BFT consensus mechanism, the system possesses instant finality, meaning that once consensus is reached, permission changes take effect immediately without waiting for multiple block confirmations as in PoW mechanisms. Consequently, in scenarios such as emergency care, doctors can obtain the latest secure authorization status in seconds.

**Comment 6:Ethical and Publication Considerations**

（1）**authors should ensure that all reused conceptual components from prior work are clearly distinguished from novel contributions.**

**Response:** We sincerely thank the reviewers for their constructive suggestions regarding clarifying distinctions in the contributions. Following this guidance, we have thoroughly revised the related work section (Section 2) to clearly distinguish reused components from our original innovations. Specifically, we have added a summary paragraph at the end of Section 2 to outline these distinctions.

（i）**Reused Components:** We explicitly state that the hierarchical access tree logic is adapted from [20, 21], the attribute revocation mechanism is integrated from [16], and the fundamental concept of BLS aggregate signatures originates from [32, 33]. Such explicit acknowledgment highlights the foundational role of the existing literature in our framework.
（ii）New Contributions: We emphasize that our BMHADS scheme fundamentally advances the underlying mathematical foundation by adopting Type-3 asymmetric pairing, thereby resolving the parameter redundancy and storage overhead issues inherent in traditional Type-1 schemes such as [20, 21]. Furthermore, we demonstrate that by introducing a DKG-based threshold signature mechanism, our scheme goes beyond the simple signature composition in [32, 33], shifting the paradigm toward decentralized consensus and thereby providing robust fault-tolerance capabilities for IoMT environments.

Once gain, we thank the editors and reviewers for their constructive comments. We believe the revised manuscript has been substantially improved and hope it is now acceptable for publication. We remain available for any further revisions.
